# Editorial: Potency assays for use in manufacturing cellular immunotherapies

**DOI:** 10.3389/fimmu.2026.1851463

**Published:** 2026-04-21

**Authors:** Donald G. Phinney, Ryang Hwa Lee, Brian H. Johnstone

**Affiliations:** 1 Department of Molecular Medicine, The Herbert Wertheim UF Scripps Institute for Biomedical Innovation and Technology, Jupiter, FL, United States; 2 Department of Cell Biology and Genetics, Vashisht College of Medicine, Texas A&M University, College Station, TX, United States; 3 Ossium Health, Inc., San Francisco, CA, United States

**Keywords:** cellular immunotherapies, cellular therapy manufacturing, immunomodulatory, potency assays, therapeutic efficacy

The clinical translation of advanced therapy medicinal products (ATMPs), including Chimeric Antigen Receptor (CAR) T cells and mesenchymal stromal cells, represents a transformative frontier in oncology and regenerative medicine. However, the successful commercialization and broad clinical adoption of these viable therapeutics are currently bottlenecked by the formidable challenge of establishing robust, predictive, and scalable potency assays. Regulatory frameworks increasingly mandate that potency metrics accurately reflect the complex, multi-factorial mechanisms of action driving a product’s biological activity. Traditional, reductionist release assays are frequently insufficient to capture the dynamic functional capacity of these “living drugs”, particularly within heterogeneous disease microenvironments or when generated via novel manufacturing procedures. This themed Research Topic underscores the critical necessity of overcoming these analytical barriers. Together, the four articles in this series proper the field away from static, single-parameter metrics and toward sophisticated, multi-dimensional, and omics-driven frameworks essential for ensuring the safety, consistency, and clinical efficacy of next-generation cellular therapies.

The series opens with an insightful review by Shao et al. who explore how the rapid evolution of CAR T-cell manufacturing demands a parallel evolution in potency assays. The authors highlight that traditional assays, which primarily measure immediate effector functions like cytotoxicity and cytokine release, are no longer sufficient. By integrating multi-omics approaches, such as genomics for vector integration sites, epigenomics for DNA methylation profiling, and metabolomics for assessing mitochondrial fitness, researchers can capture the true complexity of CAR T-cell products. Furthermore, as the field shifts toward unconventional manufacturing strategies like ultra-rapid protocols or *in vivo* CAR T-cell generation, the authors advocate for customized, omics-based potency panels to ensure product safety and efficacy.

Tackling the formidable barriers of solid tumors, Burns et al. introduce a conceptual “plying” framework for CAR-engineered platforms and tumor-infiltrating lymphocytes (TILs). Recognizing that simple 2D cell monolayers fail to recapitulate the complex, immunosuppressive tumor microenvironment, the authors propose layering potency assessments. This iterative approach progresses from regulatory-compliant release testing to advanced exploratory assays utilizing 3D tumor spheroids, label-free real-time monitoring, and spatial transcriptomics. Importantly, the integration of Artificial Intelligence (AI) and machine learning into these spatial and imaging datasets promises to uncover subtle phenotypic patterns that predict clinical performance, fundamentally transforming how we assess cellular fitness in hostile tumor microenvironments.

A highly potent cellular therapy must be safe. Li et al. provide a crucial evaluation of the systemic toxicities associated with CAR-T therapies, such as Cytokine Release Syndrome (CRS), Immune Effector Cell-Associated Neurotoxicity Syndrome (ICANS), and cardiovascular adverse events. The authors comprehensively map out predictive and diagnostic biomarkers across various organ systems. For instance, monitoring circulating free DNA (cfDNA), specific interleukins (like IL6 and IFNγ), and cardiac markers, such as hs-cTnT and NT-proNBNP, can help clinicians anticipate and mitigate severe toxicities before they become fatal. By integrating these toxicity monitoring indicators into the broader context of cellular product evaluation, this work ensures that potency does not come at the cost of patient safety.

Finally, shifting focus to regenerative and immunomodulatory therapies, Niekamp et al. tackle the longstanding issue of batch-to-batch variability in mesenchymal stem/stromal cell (MSC) manufacturing. Because MSCs modulate the immune system through multiple pathways, relying on a single surrogate marker has historically thwarted clinical success. The authors successfully developed and validated a robust quantitative matrix of potency assays for bone marrow-derived MSCs. By measuring a combination of established factors, including IDO express (for T cell suppression), M-CSF secretion (for M2 macrophage polarization), CCL2 (for monocyte recruitment) and extracellular vesicle activity, they created a weighted scoring system to reliably predict the therapeutic fitness of different MSC lots.

The collective impact of this series is profound. For the scientific community and regulatory committees, these papers provide a clear roadmap for the future of cellular therapy manufacturing. They demonstrate that the path forward requires abandoning single-parameter tests in favor of multi-dimensional, context-dependent, and AI-augmented assay matrices. By adopting these advanced analytical frameworks, researchers and manufacturers can ensure the consistency, safety, and clinical efficacy of these transformative treatments, ultimately bringing us closer to realizing the full promise of precision medicine.

